# Study on Process Optimization and Antioxidant Activity of Polysaccharide from *Bletilla striata* Extracted via Deep Eutectic Solvents

**DOI:** 10.3390/molecules28145538

**Published:** 2023-07-20

**Authors:** Liru Luo, Wei Fan, Jingping Qin, Shiyin Guo, Hang Xiao, Zhonghai Tang

**Affiliations:** 1 College of Food Science and Technology, Hunan Agricultural University, Changsha 410128, China; 13786113296@163.com (L.L.);; 2 Hunan Engineering Technology Research Center for Rapeseed Oil Nutrition Health and Deep Development, Changsha 410128, China; 3 College of Bioscience and Biotechnology, Hunan Agricultural University, Changsha 410128, China

**Keywords:** deep eutectic solvent, *Bletilla striata* polysaccharide, response surface methodology, antioxidant activity

## Abstract

Taking the extraction yield of *Bletilla striata* polysaccharide (BSP) as the index and taking the type of deep eutectic solvents (DESs), extraction time, extraction temperature, DES water content, and solid–liquid ratio as the investigation factors, single-factor and Box–Behnken response surface tests were carried out to optimize the extraction process of BSP. Thus, the antioxidant activity of BSP on DPPH radicals, ABTS radicals and ferric reducing antioxidant power were determined. The results showed that the most suitable deep eutectic solvent was DES-2, namely choline chloride-urea. The optimal extraction conditions for BSP were an extraction time of 47 min, extraction temperature of 78 °C, water content of 35%, and solid–liquid ratio of 1:25. Under this optimized condition, the extraction yield of BSP was able to reach (558.90 ± 8.83) mg/g, and recycling studies indicated the good cycle stability of the DES. Antioxidant results showed that BSP had superior antioxidant activity and had a dose–response relationship with drug concentration. Compared with *Bletilla striata* polysaccharide obtained via conventional hot water extraction (BSP-W), the extraction yield of BSP obtained through this method (BSP-2) increased by 36.77%, the scavenging activity of DPPH radicals increased by 24.99%, the scavenging activity of ABTS radicals increased by 41.16%, and the ferric reducing antioxidant power increased by 49.19%. Therefore, DESs as new green reagents and BSP extracted with DESs not only had a high yield but also had strong antioxidant activity.

## 1. Introduction

*Bletilla striata* is a dry stem of the Lango plant, mainly distributed in the wet valleys and coastal areas in the most southeastern three provinces of China. Outside China, its worldwide distribution is limited, only growing on the Korean Peninsula and in Japan, Myanmar, etc. [1]. Modern pharmacy has confirmed that polysaccharide is the main active ingredient of *Bletilla striata,* which is an effective ingredient in traditional Chinese medicine and has important medicinal value [2]. *Bletilla striata* polysaccharide (BSP) has a wide range of biological activity, with anti-ulceration [3,4], antibleeding [5], anti-inflammatory [6], antioxidant [7], antibacterial [8], antifibrosis [9] and antiaging [10] effects, for example.

At present, the methods commonly used for BSP extraction are the hot water extraction method, the acid and alkaline extraction method, the ultrasound-assisted drawing method, and the microwave-assisted draining method [11,12]. However, the hot water extraction method consumes a large amount of energy and yields are not high, while acid and alkaline extraction can damage the structure of polysaccharides. Ultrasound and microwave-assisted extraction require the use of professional instruments and are not conducive to large-scale extraction [13]. Therefore, it is critical to find a method that is inexpensive, efficient, environmentally friendly, and practical.

Deep eutectic solvents (DESs) are a new generation of environmentally friendly “green” solvents that can replace organic solvents. They have excellent potential and have attracted widespread attention [14]. Abbott et al. [15] found that amides and quaternary ammonium salts mixed in a certain molar ratio can form low-melting eutectic mixtures with unique solvent properties, and they named these DESs. DESs are ion composite solutions formed from a hydrogen key receptor and hydrogen key supplier; they have many advantages, such as low price, easy access, easy synthesis, green and environmentally friendly degradability, non-toxicity, and low volatility [16,17]. DESs have been used in organic synthesis and (biological) catalysis [18], biochemistry [19], analytical chemistry [20], nanomaterials [21], and extraction processes [22]. However, there is no report on DESs for extracting polysaccharides from *Bletilla striata*.

The purpose of this study was to create a novel method for the extraction of BSP. The study evaluated the effects of different combinations of DESs on the extraction effects of BSP. Based on the determination of the best DESs combination, the response surface test was used to optimize the core conditions parameters associated with the extraction rate of BSP and to conduct a recycling test. In addition, the antioxidant activities of the extracted BSP were determined via in vitro experiments. Comparing the yield and antioxidant activity of the *Bletilla striata* polysaccharide obtained through the best combination of DES-2 (chloride choline–urea) (BSP-2) and *Bletilla striata* polysaccharide obtained via conventional hot water extraction (BSP-W). This experiment explores a green and efficient pathway for polysaccharide extraction, providing a theoretical basis for the development of BSP.

## 2. Results and Discussion

### 2.1. DESs and Extraction Condition Screening

#### 2.1.1. Effect of Different DESs Types on the Extraction Yield of BSP

**Figure 1:**
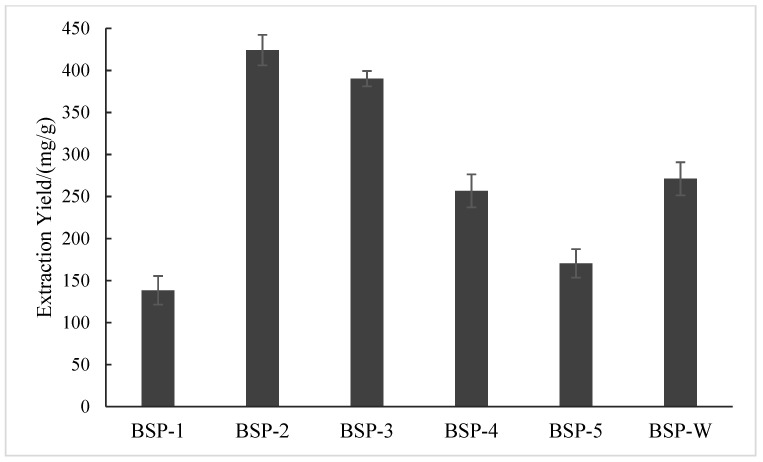
Effect of different solvent types on the extraction yield of BSP.

The effects of five DESs on the extraction yield of BSP were examined, as shown in Figure 1. Among the five DESs reagents, DES-2 (choline chloride-urea) had the best extraction effect on BSP, with the highest BSP-2 yield of 424.237 mg/g. A possible reason for this is that there is a strong hydrogen bonding ability and static interaction between DES-2 and BSP [23]. Compared with the extraction yield of BSP-W (271.035 mg/g), the extraction yield of BSP from large to small was followed by BSP-2, BSP-3, BSP-W, BSP-2, BSP-3, BSP-W, BSP-4, BSP-5, and BSP-1. The causes of the differences in extraction effects may be different in the diffusion, solubility, viscosity, polarity, and other rationalization properties between different low coefficients [24]. Based on the experimental results, DES-2 was selected for subsequent research.

#### 2.1.2. Effect of Extraction Time

**Figure 2:**
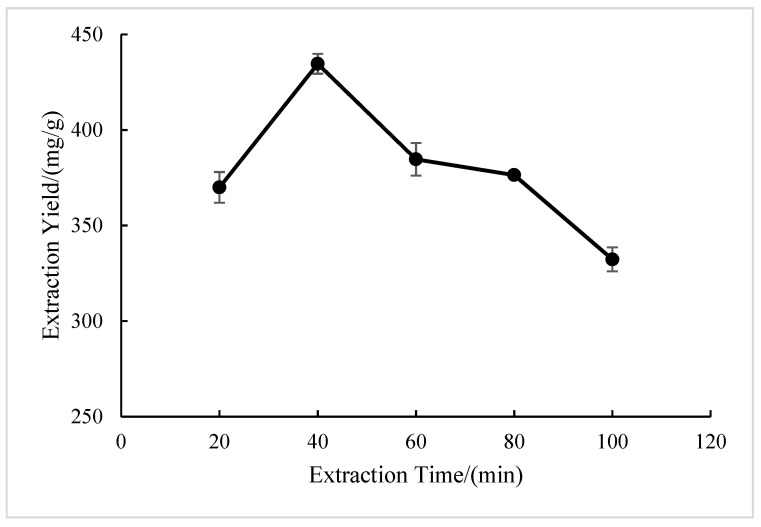
Effect of extraction time on the extraction yield of BSP.

From Figure 2, it can be seen that within 20–100 min, the extraction yield of BSP with the extension of the extraction time showed a trend of first rising and then declining, with the highest yield at 40 min. This may be due to the preliminary period with the extension time and the continuous infiltration of the extract liquid, resulting in an increase in the polysaccharide yield [25]. After reaching the peak value, the length of time was extended and polysaccharides were heated for a long time. Therefore, they were degraded, which led to a decrease in the yield rate [26]. In actual production, a reduction in the time of extraction can reduce the cost, so 40 min was chosen as the best extraction time.

#### 2.1.3. Effect of Extraction Temperature

**Figure 3:**
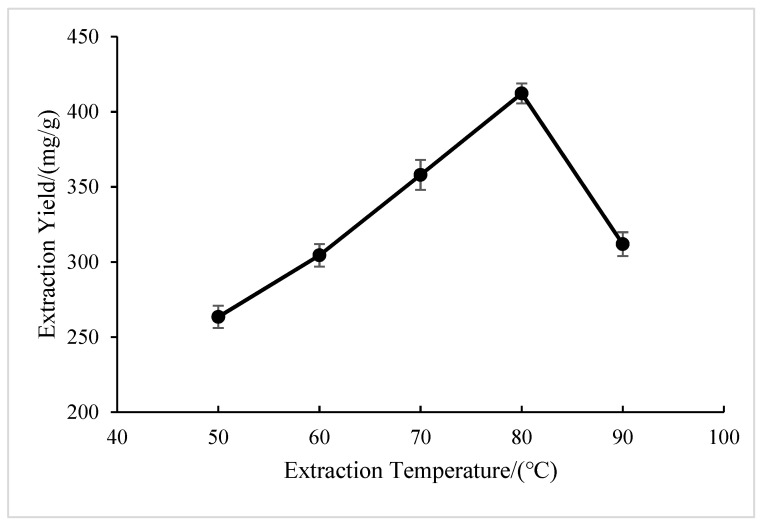
Effect of extraction temperature on extraction yield of BSP.

As shown in Figure 3, at 50 to 80 °C, the extraction yield of BSP increased as the extraction temperature rose. A probable reason could be that an increase in temperature is able to enhance mass transfer efficiency and facilitate BSP extraction [27]. BSP had a maximum extraction yield at 80 °C. Afterward, the glucose yield fell as the temperature rose. This might be the result of the polysaccharide degrading at an excessively high temperature, which lowers the polysaccharide production. Therefore, 80 °C was chosen as the ideal extraction temperature.

#### 2.1.4. Effect of DES Water Content

**Figure 4:**
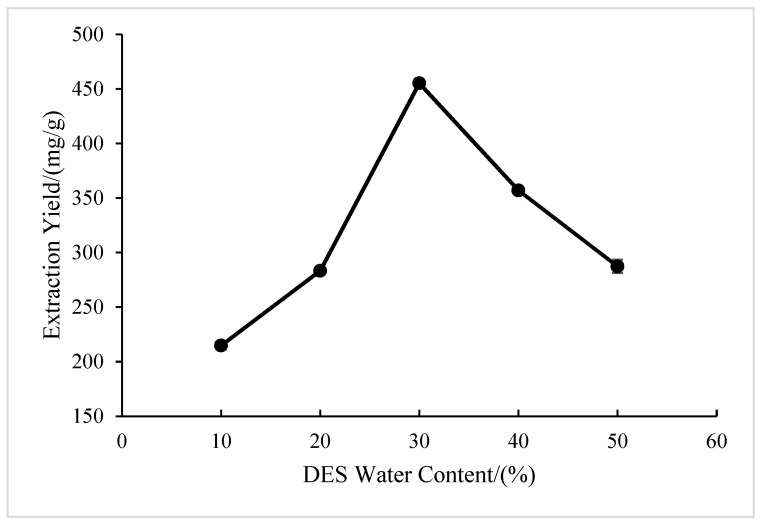
Effect of DES water content on extraction yield of BSP.

As shown in Figure 4, the extraction yield of BSP with the extension of the DES water content showed a trend of first rising and then declining, with the highest extraction yield at 30% DES water content. This could be due to the large viscosity of the DES solution system when the water content is low, which limits mass transfer. This means that polysaccharide cannot be fully precipitated, resulting in a decrease in the yield of polysaccharide [28,29]. The increase in water content can increase the dissolution rate of polysaccharides, while the viscosity of DES decreases with the higher water content. But according to the literature, the function of the hydrogen key between the hydrogen key receptor and the hydrogen key supplier is disrupted after the water-containing content exceeds 30% [30,31], so 30% was ultimately selected as the optimal water content.

#### 2.1.5. Effect of the Solid–Liquid Ratio

**Figure 5:**
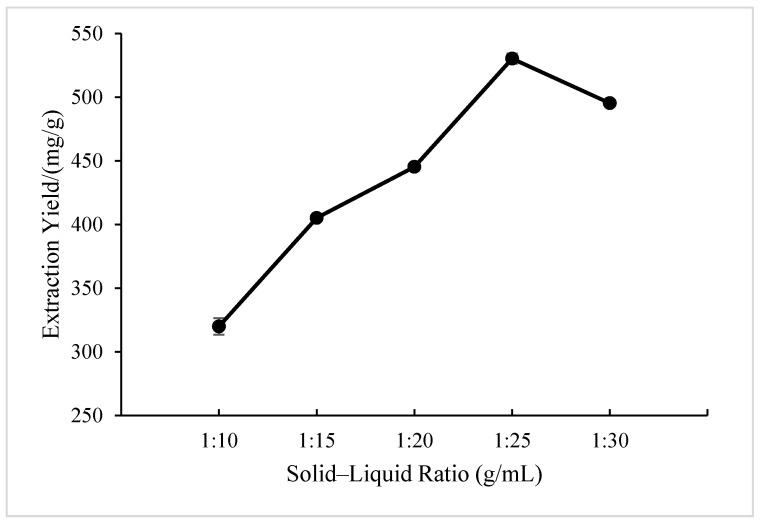
Effect of solid–liquid ratio on the extraction yield of BSP.

As seen in Figure 5, in the solid–liquid ratio of 1:10 to 1:25, the extraction yield of BSP increased with the increase in the solid–liquid ratio and reached its maximum at 1:25. However, as the dose of the solvent still increased, the BSP yield decreased slightly. This is probably because polysaccharides are fully released as the solvents increase when the solid–liquid ratio is less than 1:25. But the excessive use of solvents is not conducive to the extraction of polysaccharides, because the solute is saturated in the solvent and has an adverse impact on the mass transfer efficiency. At the same time, too many solvents can cause waste, which can also increase costs in actual production [32]. Thus, a solid–liquid ratio of 1:25 was considered the optimal choice.

### 2.2. Optimizing the BSP Extraction Conditions via the Box–Behnken Design

#### 2.2.1. Statistical Analysis and Model Fitting

On the basis of the single factor test, the extraction yield of BSP was used as the response value, with the extraction time (A), extraction temperature (B), and DES water content (C) as self-variables. According to the center-combined trial design principle of the Box–Behnken design in the response analysis method, a three-factor, three-level response surface experiment was designed. The results for a total of 17 test points are shown in Table 1.

Through regression analysis, a secondary multiple regression model of BSP extraction yield (Y) with extraction time (A), extraction temperature (B), and DES water content (C) was obtained:

Y = −7634.51500 + 22.70700A + 144.77175B + 116.96675C − 0.042100AB + 0.009330AC − 0.59785BC − 0.20991A^2^ − 0.78407B^2^ − 1.02378C^2^ (1)

#### 2.2.2. Response Surface and ANOVA

The analysis of the various variances of the regression equation is shown in Table 2.

In Table 2, it can be seen that the *p*-value was extremely low (<0.0001), which indicates that this predicted model is extremely significant [33]. The lack of fit was not significant (*p* = 0.9694 > 0.05), indicating that the fitting degree of the model was good. The coefficient of the determination (R^2^) of the variables of response was 0.9770, and the adjusted R-square (R^2^Adj) was 0.9475, which means this regression model is able to predict future results and had good credibility. The precision (C.V.%) was 6.44% (below 10%), indicating that the test had a high level of precision. Therefore, we are able to use this model to analyze and predict the extraction yield of BSP.

The monomial terms A and C and the quadratic terms BC, A^2^, B^2^, and C^2^ all reached extremely significant levels (*p* < 0.01).

**Figure 6:**
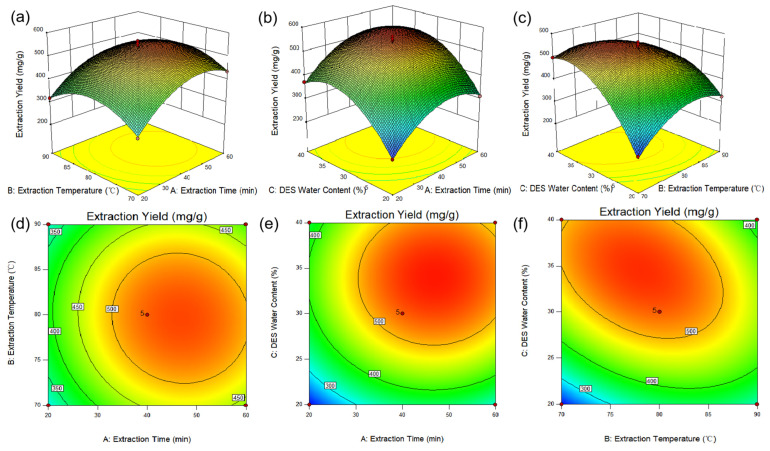
Response surface (**a**–**f**) shows the Extraction Time (A), Extraction Temperature (B) and DES water content (C) effect on the extraction yield of BSP.

The response surfaces of the interaction effects of various factors on the impact on the extraction yield of BSP are shown in Figure 6. The response surface curve of the interaction between the extraction temperature (B) and the DES water content (C) was the highest, the slope was steeper, and the high line was oval, indicating that the interaction effect between the two had the most significant impact on the extraction yield of BSP [34].

#### 2.2.3. Predicted Model Validation

Through the optimization analysis of the response surface, the optimal extraction conditions for BSP were found to be as follows: extraction time of 47.05 min, extraction temperature of 77.86 °C, and DES water content of 34.60%. Under these optimal conditions, the predicted highest extraction yield obtained was 559.66 mg/g. In order to verify the feasibility of the regression model, the above optimal operation conditions were adopted for extraction experiments. Considering the actual operation and instrument restrictions, the final extraction conditions were set to: extraction time, 47 min; extraction temperature, 78 °C; DES water content, 35%; and solid–liquid ratio, 1:25. Under these optimization conditions, the yield of BSP was (558.90 ± 8.83) mg/g, which was close to the predicted value, indicating that the experimental regression model was reliable and could more accurately predict the yield of BSP.

The use of water as an extraction reagent and other conditions were consistent with the above. The obtained BSP extraction yield was (408.63 ± 7.57) mg/g. Compared with the conventional hot water immersion method, the yield of BSP extracted by this optimization method increased by 36.77%.

### 2.3. Recycling Studies

**Figure 7:**
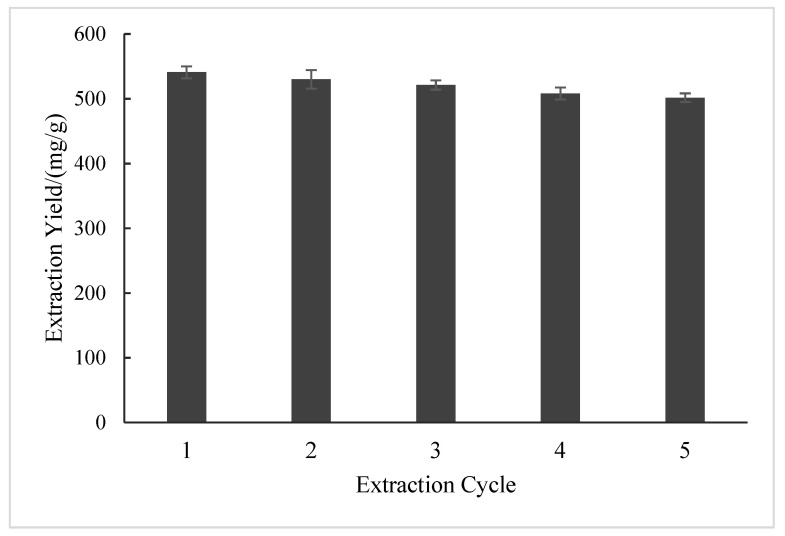
The extraction yield of BSP in the recycling tests.

BSP was extracted via the recovered DES for five cycles. As shown in Figure 7, the extraction yield of BSP declined slightly after each cycle. The extraction yields of BSP were (540.94 ± 9.15) mg/g and (501.66 ± 6.77) mg/g from the first to the fifth cycle, only decreasing by 7.26%. The decrease in extraction yield can be attributed to the introduction of impurities in the recycling process [35]. The recycling studies demonstrated that the DES could be recycled and reused well.

### 2.4. Analysis of the Antioxidant Results

#### 2.4.1. DPPH Radical Scavenging Activity

**Figure 8:**
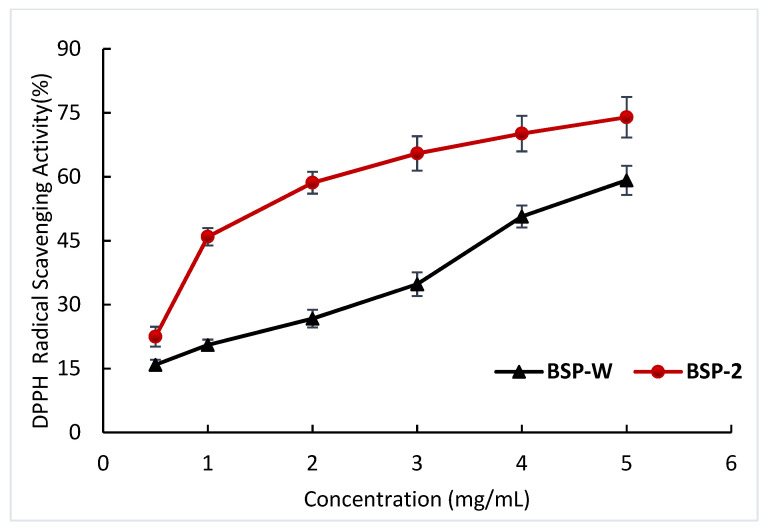
DPPH radical scavenging activities of the different BSPs.

Due to the fact that DPPH radical can form stable molecules by pairing with electrons or hydrogen radicals, the DPPH free radical scavenging assay is frequently used for evaluating the activity of natural antioxidants [36]. The DPPH radical scavenging activities of the different BSP are shown in Figure 8. The DPPH radical scavenging activities of BSP-2 increased with increased concentrations and showed a concentration–dose effect. At a sample concentration of 5 mg/mL, the scavenging activities were the largest, 73.97%, with the scavenging activities increasing by 24.99% compared to BSP-W (59.18%). The data showed that the DPPH radical scavenging activities of BSP-2 extracted with DES-2 were stronger. The reason for this is that different extraction methods that cause differences in the polysaccharide molecular weight may affect bioactivities [37,38].

#### 2.4.2. ABTS Radical Scavenging Activity

**Figure 9:**
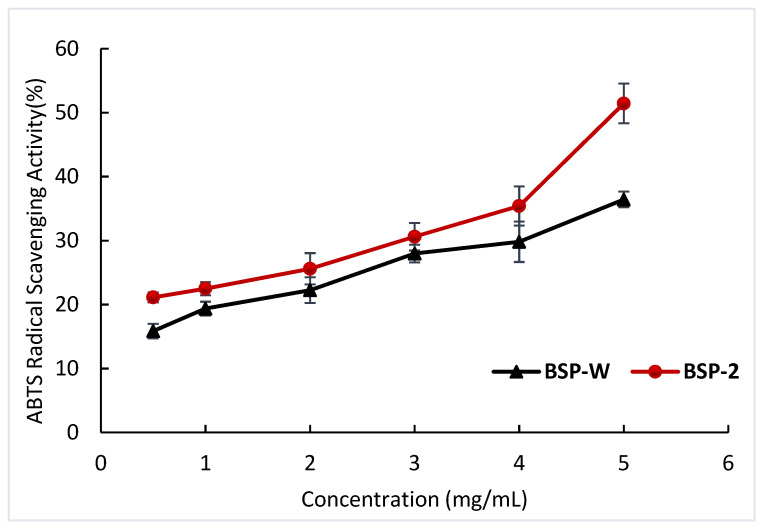
ABTS radical scavenging activities of the different BSPs.

The ABTS cation radical is formed through ABTS, peroxide and hydroperoxide. The assay for scavenging ABTS radicals is based on the decrease in absorbance at 734 nm as the ABTS radical is scavenged by antioxidant chemicals [39]. As shown in Figure 9, the scavenging activity of ABTS radical increased with increasing sample concentration, indicating that the two showed a clear dose–response relationship. At a sample concentration of 5 mg/mL, the scavenging activity was the largest, namely 51.44%. Compared to BSP-W (36.44%), scavenging activities increased by 41.16%. This indicates that the antioxidant activity of the BSP extracted from DES-2 (choline chloride–urea) was stronger than the BSP obtained using conventional hot water extraction. In addition to different molecular weights caused by different extraction methods, the cause of this result may also be that some DES components are able to improve the antioxidant activity of extracts, suggesting that there may be a synergy between DES and soluble compounds [40,41].

#### 2.4.3. Ferric Reducing Antioxidant Power

**Figure 10:**
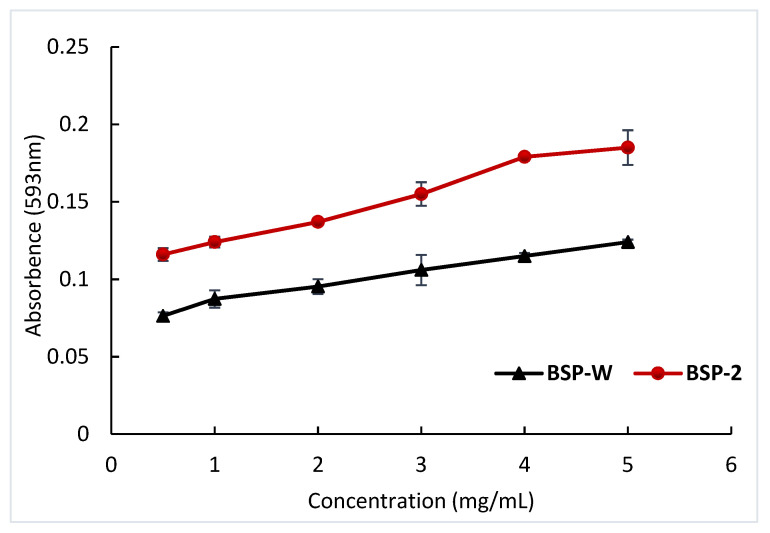
Ferric-reducing antioxidant power of the different BSPs.

The ferric reducing antioxidant power assay, which uses the antioxidant polysaccharide’s ability to donate electrons to reduce Fe^3+^ to Fe^2+^, is frequently used to assess a polysaccharide’s antioxidant capacity [42]. From Figure 10, it was shown that the ferric reducing antioxidant power was arranged from high to low: BSP-2 > BSP-W. At a sample concentration of 5 mg/mL, the ferric-reducing antioxidant power of BSP-2 was increased by 49.19% compared to BSP-W. The results showed that the BSP extracted from DES-2 was superior to the BSP extracted from hot water in terms of reducing power. Additionally, the reducing power increased as BSP-2 concentrations rose, indicating a positive correlation between the two. The different extraction methods of polysaccharides might lead to some differences in the polysaccharide structure [43]. The changes in polysaccharide structure caused by DES are probably able to improve the antioxidant activity of polysaccharides [44]. In conclusion, BSP-2 had strong antioxidant activity and was better than BSP-W.

## 3. Materials and Methods

### 3.1. Raw Materials

*Bletilla striata* was purchased from Yongzhou Prefecture of the NingYuan Prefectural *Bletilla striata* Ecological Growth Cooperative (Changsha, China). The crude materials were cleaned, cut, oven-dried at 60 °C (Electric thermostaticdrying oven GZX-9246MBE, Shanghai Boxun Industrial Co., Ltd., Shanghai, China), and crushed (Chinese herbal medicine crusher LH-08B, Chuangli Medical Machinery Factory, Wenzhou, China). The obtained powder was passed through a 40 mesh sieve to obtain the samples for further use.

### 3.2. Reagents

Choline chloride, citric acid, oxalic acid, 1,4-Butylene glycol, glycerin, absolute ethyl alcohol, sulfuric acid, and phenylphenol (all are analyzed in a pure state) were purchased from the National Pharmaceutical Group Chemical Reagents Co., Ltd. (Shanghai, China); DPPH (1, 1-diphenol-2-nitrogen), 2,4,6-three (2-phosphate) trifenol (TPTZ), and ABTS (2,2’-Azinobis-(3-ethylbenzthiazoline-6-sulphonate) (all are analyzed in a pure state) were purchased from Sigma-Aldrich (St. Louis, MO, USA).

### 3.3. Methods

#### 3.3.1. Preparation of DESs

Five DESs were chosen to extract BSP in this study (Table 3). These five DESs are representative. Compared to other DESs, the prices of these five DESs are low and the synthesis is simple. After drying the required reagent in advance, the hydrogen bond donors (HBDs) and hydrogen bond acceptors (HBAs) were weighed according to a specific molar ratio (Electronics Tianping DTY-A220, Fuzhou Huazhi Science Instrument Co., Ltd., Fuzhou, China). Then, they were mixed at 80 °C in an electric-heated thermostatic water bath (DZKW-4, ZTE Weiye Instrument Co., Ltd., Beijing, China) until most of the reagents melted [45]. Then, they were transferred to the heating magnetic stirrer (HWCL-3, Great Wall Technology Industry and Trade Co., Ltd., Zhengzhou, China) and continuously mixed to obtain a uniform liquid (DESs).

#### 3.3.2. Extraction of BSP

A total of 0.2 g of dried *Bletilla striata* powder and 3.0 mL of DESs solution (with water 30 wt%) were added to the 10 mL test tube. The mixture was placed in a 70 °C thermostatic water bath for extraction and then centrifuged (4000 rpm, 20 min). The supernatant was removed, and ethanol precipitation was used to obtain the sediment, which was oven-dried at 50 °C. The polysaccharides corresponding to DES-1, DES-2, DES-3, DES-4, and DES-5 reactors were named BSP-1, BSP-2, BSP-3, BSP-4, and BSP-5.

The sediment was dissolved with distilled water and the polysaccharide extraction yields were measured using the phenophane-sulfuric acid method [46]. The standard glucose curve was$\;y=0.007,\;x-0.0022,{\;R}^{2}=0.9952$, and the extraction yield of polysaccharide (Y) was calculated according to Formula (2):

Y/(mg·g^−1^) = CV/M (2)

where C is the polysaccharide mass concentration in the extracted liquid, mg/mL; V is the volume of the diluted extract, mL; and M is the mass of the extracted sample, g.

#### 3.3.3. Single Factor Testing

A single-factor trial was carried out using the polysaccharide yield as an indicator. The effects of the type of DESs (DES-1, DES-2, DES-3, DES-4, and DES-5), extraction times (20, 40, 60, 80, and 100 min), extraction temperatures (50, 60, 70, 80, and 90 °C), DES water contents (10 wt%, 20 wt%, 30 wt%, 40 wt%, and 50 wt%), and solid–liquid ratios (1:10, 1:15, 1:20, 1:25, and 1:30) on the extraction yield of BSP were examined, respectively.

#### 3.3.4. Response Surface Test

Based on the results of single-factor testing, the response surface test was used to optimize the extraction process. The Box–Behnken design was conducted using the Design-Expert 10.0.1 software. The three-factor (A: extraction time; B: extraction temperature; C: DES water content), three-level response surface experiment was designed and the changes in the polysaccharide yield were examined. Regressive analysis based on experimental data. Subsequently, three validation extraction trials were conducted under optimal conditions to verify the accuracy of the statistical test strategy. The factor level design is shown in Table 4.

#### 3.3.5. Recycling Test

To explore the retrievability of the DES, the DES solution that was precipitated by ethanol was taken, and the ethanol was removed using a rotary evaporator (RE-200B, Zhongtian Science and Technology Instrument Co., Ltd., Gongyi, China) to obtain the recovered DES. The BPS was extracted with the recovered DES solution. This process was repeated five times.

#### 3.3.6. Antioxidant Test

##### DPPH Radical Scavenging Activity

Following the Slimkard [47] methodology with slight modifications, 100 μL of polysaccharide solution of different concentrations and 100 μL of 0.1 mmol/L of DPPH ethanol solution (now available) were added to 96 porous plates. Then, they were mixed on the microoscillator (MH-2, Qiliinbel Instrument Manufacturing Co., Ltd., Haimen, China). They were placed in a dark place at 25 °C for 0.5 h, and then the absorbance was measured at 517 nm in the multifunctional enzyme label instrument (En Spire, Polkin Elmer Instrument Co., Ltd., Waltham, MA, USA). Formula (3) was used to calculate the clearance rate of the DPPH free radicals.

DPPH Scavenging Activity (%) = [1 − (A_i_ − A_j_)/Ao] × 100% (3)

where A_i_ is light absorbance after sample reaction balance; A_j_ is the sampling itself (the sample + 95% ethanol); and Ao is the non-added sample’s DPPH free-radical absorbance (95% ethanol + DPPH–ethanol solution).

##### ABTS Radical Scavenging Activity

A total of 2.45 mmol/L of manganese sulfate solution and 7 mmol/L of ABTS solution were mixed in equal proportions and were left overnight for 12 h to obtain the ABTS mother liquid. The ABTS mother liquid was diluted with 80% ethanol, and the absorbance was measured to approximately 0.70 at a wavelength of 734 nm. A total of 40 μL of polysaccharide solution and 160 μL of ABTS dilution liquid were added to 96 porous plates. They were mixed on the MH-2 micro-oscillator and left at room temperature for 8 min. Absorbance was measured at 734 nm. Formula (4) was used to calculate the clearance rate of the ABTS free radicals.

ABTS Scavenging Activity (%) = [1 − (A_i_′ − A_j_′)/Ao′] × 100% (4)

where A_i_′ is light absorbance after sample reaction balance; A_j_′ is the sampling itself (the sample + 80% ethanol); and Ao′ is the non-added sample’s DPPH free-radical absorbance (80% ethanol + ABTS–ethanol solution).

##### Ferric-Reducing Antioxidant Power

The ferric-reducing antioxidant power test method follows the Pulido [48] method with slight modifications. First, the 0.3 mol/L sodium acetate buffer, 20 mM FeCl_3_·6·H_2_O solution, and 10 mM TPTZ (with a 40 mM HCL configuration) were mixed in at a 10:1:1 ratio to be stored at 37 °C conditions, prepared as a FRAP working liquid. A total of 10 μL of different concentrations of polysaccharide solution or FeSO_4_ standard solution (10 to 300 μM) were added to 96-well porous plates. Then, 200 μL of FRAP working liquid and 10 μL of ultra-pure water were added. They were mixed on the MH-2 micro-oscillator and placed in the water boiler at 37 °C for 8 min. The absorbance was measured at 593 nm with the En Spire multifunctional enzyme label instrument. The standard curve was made using the FeSO_4_ solution. The corresponding FeSO_4_ concentration was obtained from the curve when the same absorption of light. The return value was expressed via the appropriate FeSO_4_ concentration (μmol/L).

#### 3.3.7. Statistical Analysis

The responsive test data were compiled and statistically analyzed using Design-Expert.10.0.1 software in this trial. And the data related to single-factor trials and antioxidant trials were analyzed using the Origin 2021 software. Each trial was parallel measured 3 times, and the results are given as the averages.

## 4. Conclusions

This article established a process for high-efficiency, green DESs to extract BSP. By studying the effects of different combinations of DESs on the extraction yield of BSP, DES-2 (choline chloride:urea = 1:2) was selected as the optimal extractant. The extraction conditions were optimized by single factors and BBD as follows: extraction time of 47 min, extraction temperature of 78 °C, DES water content of 35%, and solid–liquid ratio of 1:25. The extraction yield of BSP under these ideal conditions was (558.90 ± 8.83) mg/g, 36.77% higher than that of BSP-W. And recycling studies indicated that the DES-2 could be recycled and reused well, while the extraction yield of BSP only decreased by 7.26% after the fifth cycle. In addition, compared to BSP-W, the polysaccharides obtained through DES-2 showed a higher DPPH radical scavenging activity, ABTS radical scavenging activity, and ferric-reducing antioxidant power, which indicated that the polysaccharides obtained via this method (BSP-2) had stronger antioxidant activity. In summary, utilizing DESs to prepare BSP was able to increase both antioxidant activity and extraction yield, and the BSP ingredient extracted from DESs was able to maintain good bioactivity. It is known that the process can be used for the extraction of natural polysaccharide components, laying the foundation for further applications in food and medicine.

## Figures and Tables

**Table 1:** Response surface test design and results.

Number	A: Extraction Time/min	B: Extraction Temperature/°C	C: DES Water Content/%	Extraction Yield /(mg·g^−1^)
1	60	80	20	313.47
2	60	90	30	420.78
3	40	80	30	564.16
4	40	70	20	219.00
5	20	80	40	371.69
6	40	90	20	323.44
7	40	90	40	361.42
8	60	80	40	483.01
9	40	80	30	547.49
10	20	70	30	299.20
11	40	80	30	553.20
12	40	70	40	496.12
13	20	80	20	209.59
14	20	90	30	319.18
15	60	70	30	434.48
16	40	80	30	493.84
17	40	80	30	495.21

**Table 2:** Results of the ANOVA.

Source	Sum of Squares	df	Mean Square	F-Value	*p*-Value	
Model	20,370	9	22,633.76	33.11	<0.0001	Significant
A	25,547.04	1	25,547.04	37.37	0.0005	
B	71.88	1	71.88	0.11	0.7552	
C	52,284.08	1	52,284.08	76.47	<0.0001	
AB	283.59	1	283.59	0.41	0.5401	
AC	13.84	1	13.84	0.020	0.8909	
BC	14,296.98	1	14,296.98	20.91	0.0026	
A^2^	29,682.95	1	29,682.95	43.42	0.0003	
B^2^	25,885.20	1	25,885.20	37.86	0.0005	
C^2^	44,131.17	1	44,131.17	64.55	<0.0001	
Residual	4785.74	7	683.68			
Lack of Fit	259.85	3	86.62	0.077	0.9694	Not significant
Pure Error	4525.89	4	1131.47			
Cor Total	208,500	16				
R^2^	0.9770					
R^2^Adj	0.9475					
C.V.%	6.44					

**Table 3:** Synthesis and molar ratios of the solvents of the different DESs.

Number	HBAs	HBDs	Molar Ratio
DES-1	Chloride choline	1,4-Butylene glycol	1:4
DES-2	Chloride choline	Urea	1:2
DES-3	Chloride choline	Oxalic acid	1:1
DES-4	Chloride choline	Citric acid	1:1
DES-5	Chloride choline	Glycerin	1:2

**Table 4:** Design and level of response surface factors.

Level	Factor		
	A: Time/min	B: Temperature/°C	C: DES Water Content/%
−1	20	70	20
0	40	80	30
1	60	90	40

## Data Availability

Data are contained within the article.
